# Synthesis of monolith silica anchored graphene oxide composite with enhanced adsorption capacities for carbofuran and imidacloprid

**DOI:** 10.1038/s41598-022-25528-0

**Published:** 2022-12-05

**Authors:** Musa Khan, Mian Muhammad, Zeid A. AlOthman, Won Jo Cheong, Faiz Ali

**Affiliations:** 1 Department of Chemistry, University of Malakand, Chakdara, Lower Dir, 18800 Pakistan; 2 Department of Chemistry, College of Science, King Saud University, 11451 Riyadh, Saudi Arabia; 3 Department of Chemistry, Inha University, 100 Inharo, Namku, Incheon, 402-751 South Korea

**Keywords:** Environmental sciences, Chemistry, Materials science

## Abstract

Highly efficient adsorbent was prepared for the removal of carbofuran and imidacloprid pesticides from wastewater. The silica monolith anchored graphene oxide composite was synthesized by the modified Fischer esterification protocol. The composite showed improved adsorption capacity for the removal of pesticides from wastewater. Graphene oxide was synthesized using the modified Hummer’s method, while the silica monolith was prepared via sol–gel method. The composite was characterized via X-ray diffraction, Fourier transform infra-red, Brunauer Emmett and Teller (BET/BJH) analysis, zeta potential, and FESEM imaging. Different adsorption parameters such as pH, contact time, adsorbate and adsorbent concentration, and temperature were optimized for the adsorption of pesticides. The equilibrium and kinetic models were applied to the adsorption process of the pesticides. Qe of the composite as found to be 342.46 mg g^−1^ for imidacloprid and 37.15 mg g^−1^ for carbofuran. The adsorption process followed the pseudo 2nd order kinetic model for carbofuran (*R*^2^~0.9971) and imidacloprid (*R*^2^~0.9967). The Freundlich isotherm best fitted to the adsorption data of the pesticides with *R*^2^ value of 0.9956 for carbofuran and 0.95 for imidacloprid. The resultant adsorbent/composite material came out with very good results for the removal of pesticides.

## Introduction

Since the nineteenth century, different chemical products have been using for the plant protection in agriculture, which are collectively known as pesticides^1^. According to the World Health organization (WHO), any chemical compound which is used for plant protection by killing the pests are called “pesticides”. It was noticed that those pesticides have serious effects on air, soil, and water. According to the needs of world population, use of such pesticides for the high yield production of good quality foods is unavoidable fact. The use of pesticides can lead to serious environmental problems and health hazardous issues^2^. Acetylcholine produces impulse transmission via enhanced hydrolysis by the action of Acetylcholinesterase (AChE) in nervous system. The inactivation of AChE enzyme by the pesticides can cause hyperstimulation of muscarinic and nicotinic receptors resulting in the blockage of the neurotransmissions^3^.

**Figure 1:**
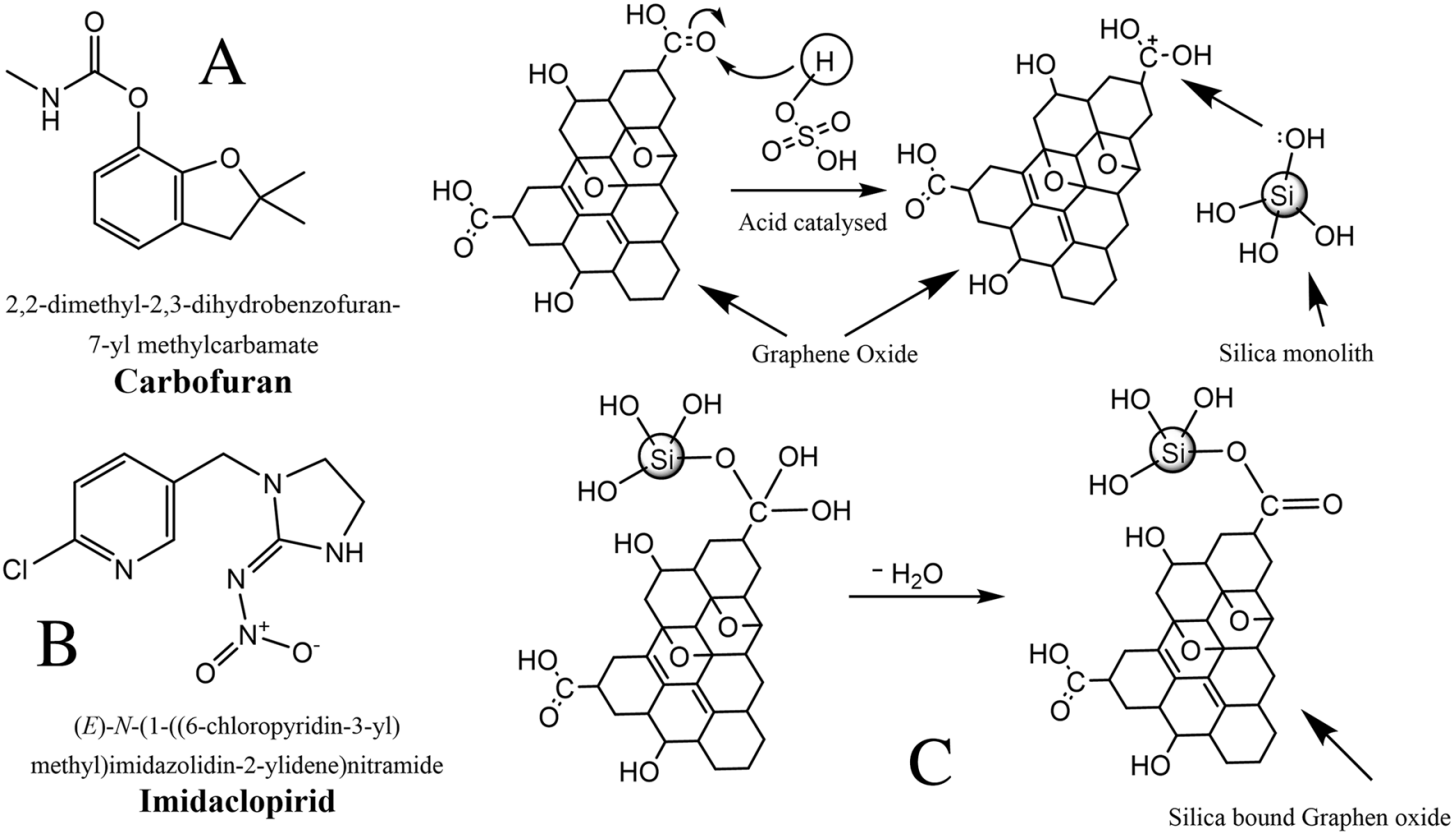
Structures of carbofuran (**A**) and imidacloprid (**B**), Scheme for the Synthesis of composite availing the Fisher Esterification protocol (**C**).

Carbofuran is a carbamate insecticide used for the control of nematodes and other insects in vegetable, fruit, and crops. It has a negative effect on the human reproductive system^4^. The second mostly used pesticide for the quality and quantity improvements of the crop is imidacloprid. Due to long lifetime, small particles size, and higher solubility it is hazardous for aquatic environment. imidacloprid leach out to the surface in water environment and remain in the food for longer time^5^. Structures of carbofuran and Imidacloprid are given in Fig. 1.

Various techniques are in action for the removal of pesticides from wastewater such as oxidation, degradation, photo Fenton, s technique, and membrane technology^6,7^. The advanced oxidation process (AOP) is one of the effective, easiest, and most commonly used method applied for the removal of organic pollutants, but its separation capability is limited to the type and nature of the adsorbents^8^. In Fenton process hydroxyl radical is released by the breakdown of water molecule which oxidizes the organic pollutants. The Fenton process in the presence of visible or UV light is called photo-Fenton. The generation of sludge during post treatment, metal ion discharge and limited pH range are the disadvantages of the Fenton process^9^. The photocatalytic oxidation process is the more versatile technique in which the light excite electron from ground level in molecules, which further oxidizes the organic pollutants in water. The major disadvantages of this process are limited quantum efficiency owing to the less intensive visible light and deactivation of the photocatalyst^10^. In ion exchange method, a particular ion in liquid must be removed by exchanging the ion with that of the solid phase. But nevertheless, the contaminant in wastewater mostly affects the process^11^.

Adsorption is one of the effective, and simple technique used for wastewater treatment using various adsorbents^12^. Towards the elimination of pesticides/pollutants from wastewater the Adsorption is an effective technique in which the pollutants bind at the adsorbent surface^7^. Adsorption technique offers many advantageous features including improved performance, operational ease, employment of the low-cost adsorbent materials/inexpensive, and stability. Other techniques are associated with one or the other limitation listed above. It reduces the pollutants concentration to very low level^13^.

To synthesize the multifunctional and more efficient adsorbents for the removal of organic pollutants from wastewater the selective combinations of raw materials can be used. Graphene oxide has OH, COOH and COC functionalities on its surface which can form different types of bonds with silica monolith where the latter possesses the silanol groups on its surface. Depending on the functional groups and nature of organic pollutants the chemistry of graphene oxide silica monolith adsorbent can be fine-tuned. The unmodified graphene oxide is unstable which makes its handling/recovery after use very difficult, and it blocks the pores of filter paper causing issues in filtration and centrifugation. After the incorporation of silica monolith into its network increases its stability and it can easily be handled^14^. Silica-based nanocomposite are aqua friendly, physiochemically stable materials which can be used as good adsorbent for the removal of pesticides^15^. Large pore size, large surface/volume ratio of the silica-based materials, enhance adsorption capability towards pesticides make it a good choice for adsorption. Silanol groups on the silica surface make it capable to form a variety of composite with other materials^16^. Metal oxide nanoparticles/iron oxide may be released from graphene oxide sheets binding via electrostatic interaction and physical adsorption. To overcome this problem silica-based materials can be applied to the GO, that can easily be dispersed and separated^17^. The *Esterification* reaction of the graphene oxide with amine and cyanate has been reported. Similarly, the amine functionalities of the chitosan are responsible for the esterification reaction with carboxylic group of GO^18^. Silica monolith and its modified composite materials can be used as the best adsorbents^19^. In current study we demonstrated the synthesis of silica monolith anchored graphene oxide composite using silica monolith and graphene oxide by Fischer esterification protocols. The composite materials were applied for the removal of carbofuran and imidacloprid from wastewater with highly improved adsorption capabilities.

## Materials and methods

### Chemicals and reagents

All the chemicals were of analytical grade. Imidacloprid (Sigma Aldrich, Mol.wt-255.66 g mol^−1^) and carbofuran sigma Aldrich Mol. Wt-221.25 g mol^−1^) were used without further purification. Urea, Glacial acetic acid, polyethylene glycol (PEG), were purchase from Sigma Aldrich. HCl (37%), NaOH, NaNO_3_, KMnO_4_ and H_2_O_2_ were purchased through local vendors from Sigma Aldrich, Germany. Briton Robinson buffer reagents, H_3_PO_4_, H_3_BO_3_ (China) and CH_3_COOH (Sigma Aldrich Germany) were used for the adjustment of desire pH.

### Instrumentation

The Spectrophotometer-1601 Shimadzu Japan with glass cell of 1.0 cm path length was used for the measurement of absorbance against blank solvent. The stirring hot plate (Irmico, Germany) 280–500 ℃, 0–2000 rpm, was used for heating and stirring. The furnace (Nabertherm, Germany) was used. The composite centrifugation from the aliquot was carried out using centrifuge (Witeg, Germany). pH readings were taken with pH meter (Hanna, USA), and the sonicating machine (Power sonic-405, Korea) was used for sonication. For shaking purposes, orbital shaker (Taiwan) was used.

### Characterization techniques

The resultant composite was characterized by the Brunauer, Emmett and Teller (BET) analysis, Fourier transform infra-red (FTIR), X-ray diffraction (XRD), zeta potential (zeta pH), and FESEM imaging. The adsorption of gases on the composite surface is the most versatile techniques for the exploration of the surface area and pores size distribution of the porous composite. BET Pores distribution and specific surface area of the composite were measured using the Mesh No. 16 [ASTM E:11] via an instrument of volumetric gas adsorption which uses the nitrogen gas as adsorptive (Quanta chrome NOVA 2000 e series USA). Adsorption experiments were carried out at 75.4 K and p/p_o_ = 0.96. nitrogen gas. The moisture adsorbed on the composite surface were evaporated under vacuum pressure at 100 °C for 8 h before the calculation of pore size distribution, and specific surface area of the composite^20^. The FTIR study of the composite was carried out by using Excalibur spectrometer with standard detector. Various functional groups on the surface of silica anchored GO composite, Silica monolith and GO surface, KBr pellets were applied. The resultant spectra were documented with resolution of 4 cm^−1^ taken in the region of frequency corresponding to 4000 to 400 cm^−1^^21^. Zeta potential was measured via nano trac II zeta analyzer instrument, using composite suspension in water. The sample was taken in zeta cell containing a small line called front. Before taking the sample, the cell was cleaned with lens paper. The cell was placed in instrument (nano trac II zeta analyzer instrument, USA). The analysis was performed at a temperature 0.4 ℃ and equilibrium time of 3 min, and at a manual applied voltage of 100 nV^22^.

### Synthesis of monolith silica particles

The silica monolith particles were prepared using the sol–gel protocol (1650 mg urea and 1620 mg PEG were dissolved in 15 ml acetic acid solution (0.01 N) in a Teflon vial. The solution was stirred magnetically for 10 min in ice cold environment. 5.00 ml of the Trimethoxy silane (TMOS) was added to the mixture, and stirred magnetically for 40 min. The mixture was dried at 40 °C in Lc oven for 40 h, then at 125 °C in an autoclave for further 45 h using a GC oven. The resultant residual water form due to sol–gel process was removed and the resultant monolithic silica was vacuum dried at 60 °C for 25 h. The resultant silica monolith was grounded up with a mortar and pestle for 10 min, and finally calcined at 500 °C in a muffle furnace^23^.

### Synthesis of graphene oxide sheets

Graphene oxide sheets were prepared by Hummer method. A mixture of graphite powder (3.00 mg), sodium nitrate (2.00 mg) in 70 ml H_2_SO_4_ (98%) was taken in 500 ml two neck round bottom flask at a temperature bellow 20 °C. The solution was stirred for 3 h after the addition of KMnO_4_ (9.00 gm). The solution was stirred for 3 h after keeping it in oil bath at a temperature of 40 °C. 50 ml distilled water was added to the mixture in a drop wise manner at a temperature of 35 °C and a color change from dark to yellow was observed. 100 ml water was added again, and the solution was kept on stirring for 15 min at 95 °C. 200 ml H_2_O_2_ (30%) was added to the mixture and stirred for 2 h. The resultant solid GO was cooled, filtered, and washed with 1:10 HCl and water mixture and dried in air^24^.

### Synthesis of Silica monolith-graphene oxide composite

The silica monolith@Graphene oxide composite was synthesized using graphene oxide and silica in 1:3. Graphene oxide (0.1 g) was dispersed in 100 ml distilled water and sonicated for 30 min similarly, the 0.36 g silica was dispersed in water. The two suspensions were mixed in the above given ratio in a round bottom flask. 1.0 ml H_2_SO_4_ (98%) was added to the solution. The entire mixture was reacted at 100 °C for 24 h under reflux condition^25^. The schematic synthesis of the composite is shown in Fig. 1.

### Batch adsorption studies

The adsorption capabilities of the composite were studied for the adsorption of carbofuran and imidacloprid in wastewater. Various adsorption parameters such as contact time, temperature effect, adsorbent and adsorbate dose, and pH effect were optimized, using a specific amount of the composite and pesticides (imidacloprid and carbofuran)^26^. During the adsorption studies, 3 mg composite was added to 20 ppm solution of the imidacloprid at optimized pH ~ 5). 6 mg of the composite was used for the adsorption of carbofuran (20 ppm) solution at optimize pH ~ 2). All the solutions for both pesticides were placed on orbital shaker for the optimization of contact time. The absorbances of all the solutions were recorded spectrophotometrically at their maximum wavelength of absorbance (269 nm for imidacloprid and 275 nm for carbofuran). The absorbance capacities and % adsorptions were calculated using the following equations.

$$\begin{aligned} qe & = \frac{{\left( {Ci - Ce} \right)V}}{W} \\ \% Adsorption & = \left( {Ci - Ce} \right)/Ci \times 100 \\ \end{aligned}$$

where Ci and Ce are the pesticides concentration in (mg L^−1^) in liquid solution. W is the weight of adsorbent (mg g^−1^), while V is the volume of liquid in (L).

The amounts of pesticides both absorbed and unabsorbed were calculated and the percent adsorptions of pesticides were finally reported at the optimized conditions of adsorptions.

### Isotherm and kinetic studies

In adsorption isotherm studies, the silica anchored graphene oxide composite (6 g/L ⁓ imidacloprid and 10 g/L ⁓ carbofuran) were added to 50 ml flasks containing pesticides solutions of different concentration (10 to 50 ppm) at optimized pH, (imidacloprid pH 5, carbofuran pH 2). The composite and pesticide solutions were mixed at 200 rpm at room temperature for 160 min and the concentration of each pesticide in aliquot was determined. The kinetic studies of the pesticides adsorption were carried out using the optimized conditions for other parameters using the different time intervals (0–200 min).

## Results and discussion

### Fourier transform infra-red spectroscopy

**Figure 2:**
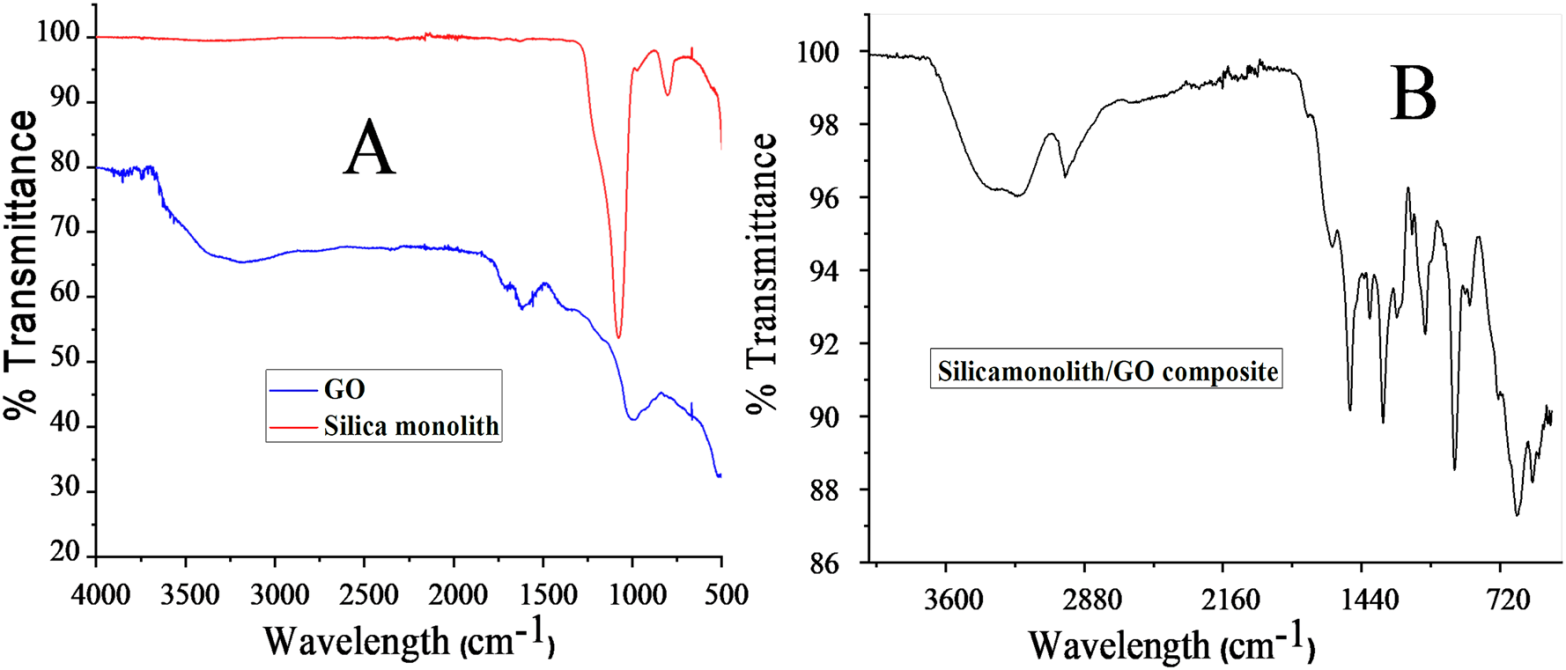
FTIR images of graphene oxide and silica monolith (**A**): silica bound graphene oxide composite (**B**).

The FTIR spectrum for silica monolith, graphene oxide, and silica anchored graphene oxide are given in Fig. 2 to showcase the different functional groups. *FTIR spectra of GO*: the broad peak at 3600–3000 cm^−1^ shows the stretching frequency of OH group. The peak at 1616 cm^−1^ represents C=C. The 1718 cm^−1^ peak represent the C=O group. The C–O functional group is represented by the peak at 975–1020 cm^−1^^27^ as shown in Fig. 2A. *FTIR spectra of silica monolith*: the sharp peaks at 1111 to 1188 cm^−1^ show the stretching frequency of Si–O–Si functional group. The small appearance of a broad peak at 3400 cm^−1^ is due to the stretching frequency of OH group^28^. *FTIR spectra of silica anchored graphene oxide:* the peak at 1020 cm^−1^ and 1100 cm^−1^ represents the stretching frequency of C–O. The disappearance of peak at 1735 cm^−1^ for carboxyl group on graphene oxide and appearance of peak at 1380 cm^−1^ attribute to the vibrational frequency corresponding to Si–O–C=O of the siloxane networking formed by the reaction of COOH group of GO and Si–OH of silica monolith during the composite synthesis. The peaks at 550 and 650 cm^−1^ show the stretching frequency of Si–O of silanol group in the composites. The peak at 2980 cm^−1^ corresponds to the presence of CH_2_ group on the surface of graphene oxide. The broad peak at 3100–3400 cm^–1^ represent OH group and hydrogen bonding^29^. These changes in the FTIR spectrum confirm the successful formation of GO-Silica monolith composite as shown in Fig. 2B.

### XRD analysis

**Figure 3:**
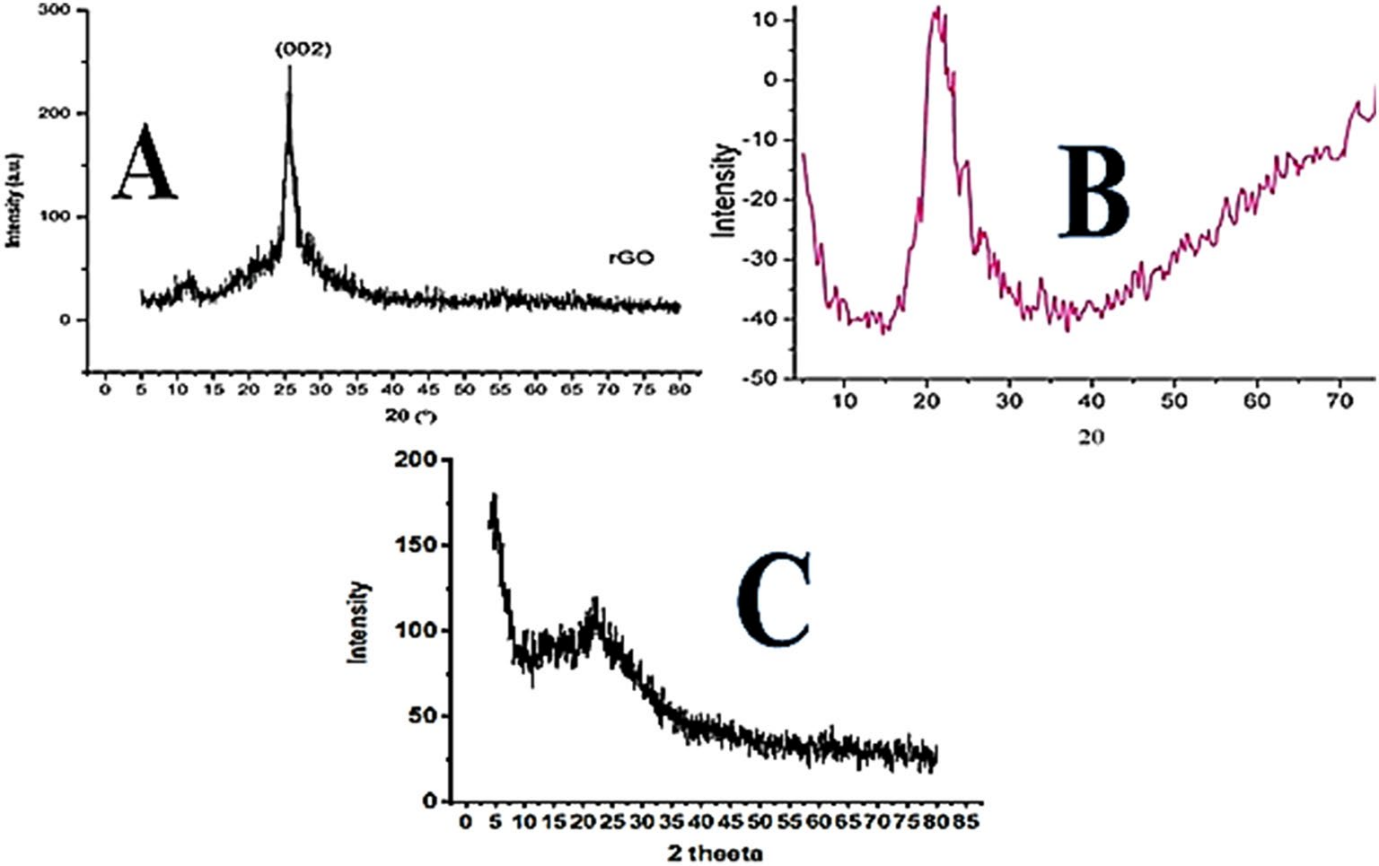
XRD pattern of Graphene oxide (**A**), Silica particles (**B**), and silica modified graphene oxide composite (**C**).

In the XRD pattern of graphene oxide a diffraction line (002, d-spacing 0⋅34 nm at 26⋅05°) shows that the GO was partially oxidized as shown in Fig. 3A. A specific peak for graphene oxide formed at 10.89° (d-spacing between sheets of 0.81 nm) is due to the presence of hexagonal arrangement of carbon in sheet to form the graphene oxide^30^. The same results can be observed in the XRD pattern of the graphene oxide which was synthesized by the Hummer’s method^31^. In the XRD image of silica monolith a broad intense peak at 20°–23° attribute to Si–OH and OH on the surface of silica monolith as shown in Fig. 3B^32^.

The XRD pattern of silica anchored GO confirms the presence of both phases (GO and silica monolith) in the composites. The intense peaks at 2θ = 0.4°–2.4° up to 100–200 diffraction peaks show two dimensional well order hexagonal structure present in the composite of silica anchored GO^33^. The broad peak at 20°–23° is due to silica, while the peak at 11° and 26° peaks are due to GO as shown in Fig. 3C. The XRD of the silica anchored GO samples has a slight shift in the (002) peak maximum from 26⋅05° in GO to 26⋅20° which may be due to the reduction of GO sheets^34^. These reports of XRD represent and confirms the successful formation of silica monolith anchored GO composite by the renovated Fisher esterification protocol.

### Analysis by Brunauer, Emmett and Teller (BET/BJH)

**Figure 4:**
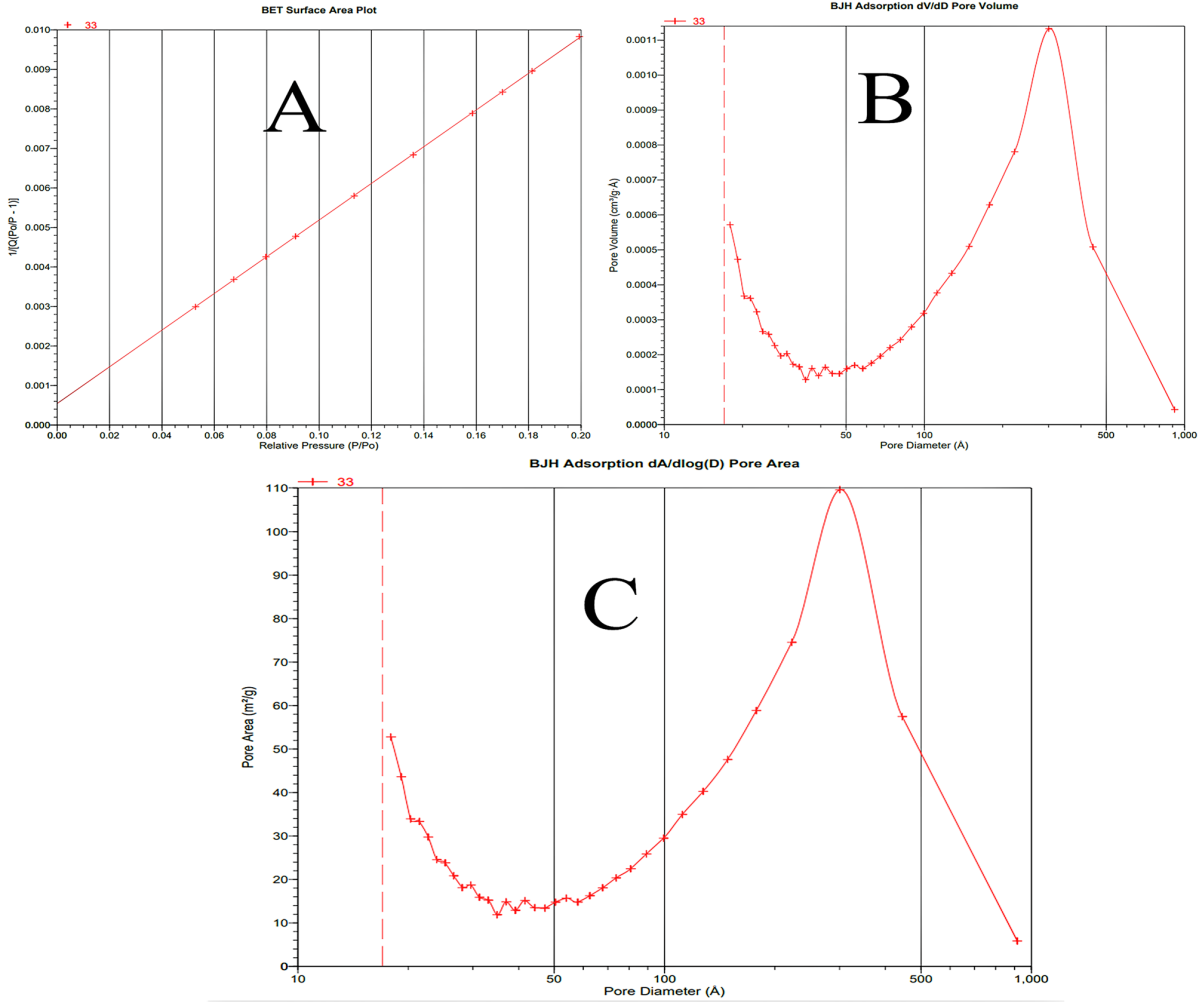
The BET surface area (**A**), BJH adsorption (dV/dD) pore volume (**B**), and BJH adsorption dA/dlogD pore area (**C**) of the composite.

BET/BJH analysis explains the specific surface area and porosity of the newly synthesized silica monolith graphene oxide composite. The BET/BJH plots are given in Fig. 4 and the analysis summery is given in Table 1. The composite showed specific surface area of 274.5 (m^2^/g), pore volume 0.095 cc/g and pore radius of 135.492 Å. The higher surface area of the composite shows that specific cavities/pores are formed for better adsorption of the analyte. Porosity of a particle represent the volume ratio of open pore to the total volume^35^. The greater adsorption capability of the composite is due to the high surface area^36^. Furthermore, the higher loading capacity and higher throughput analysis of the composite is due to the higher total volume^37^. The average dimeter of pore was reported in the range of 2–5 nm, showing the mesoporous nature of the composite^38^.

**Table 1:** BET/BJH analysis summary.

	Reaction conditions/descriptions	Results of summary report
Surface area (m^2^/g)	Single point surface area at P/Po = 0.199472855	289.678922 (m^2^/g)
	BET surface area	296.199876 (m^2^/g)
	t-plot micropore area	16.2891 (m^2^/g)
	t-plot external surface area	273.2873 (m^2^/g)
	BJH adsorption cumulative surface area of pores between 17.000 Å and 3000.000 Å diameter	261.7893 (m^2^/g)
	BJH Desorption cumulative surface area of pores between 17.000 Å and 3000.000 Å diameter	294.5432 (m^2^/g)
Pore volume (Cm^3^/g)	Single point adsorption total pore volume of pores less than 2566.419 Å diameter at P/Po = 0.992420088	0.520998 (Cm^3^/g)
	t-plot micropore volume	0.002496 (Cm^3^/g)
	BJH adsorption cumulative volume of pores between 17.000 Å and 3000.000 Å diameter	0.502260 (Cm^3^/g)
	BJH desorption cumulative volume of pores between 17.000 Å and 3000.000 Å diameter	0.521090 (Cm^3^/g)
Pore size (**Å**)	Adsorption average pore width (4 V/A by BET)	224.8209 (Å)
	BJH adsorption average pore diameter (4 V/A)	263.227 (Å)
	BJH desorption average pore diameter (4 V/A)	226.691 (Å)

### Zeta potential

Zeta potential of the composite is related to the point of zero charge. The value of pH at which the net surface charges of the adsorbent become zero is called the point of zero charge (pzc). The adsorption sites on the adsorbent are charged surfaces due to the deposition of proton or hydroxide ions at different pH. The negative zeta potential at pH values of less than 7 showed that the protons are deposited on the surface of the adsorbent and become cationic in nature. Thus, the negative surface can easily adsorb the anionic species. Similarly, in basic media the deposition of OH ions are difficult on the adsorbent surface. The negative zeta potential at pH values higher than 7 is due to the protonation of hydrogen from adsorbent silanol group to the hydroxide ions in solution or complex formation^39^.

Zeta potential is used for characterizing the electrical properties of interfacial layers in dispersion^40^. The negative zeta potential values are due to the presence of electronegative functional groups formed at the graphite lattice during the oxidation process. With successive increase in the oxidation density, a greater number of electronegative functional groups are formed in GO resulting in the higher magnitude of the zeta potential at higher oxidation levels. More oxygenated functional groups with a higher zeta potential in an aqueous medium is more likely due to the dissociation of a greater number of acidic groups (COOH → COO^−^ + H^+^) at the surface thereby resulting in a higher zeta potential. According to the American Society for Testing and Materials( ASTM ) standards for stability of colloidal suspensions, a zeta potential less than 30 mV shows less stability, potential between 30 and 40 mV (either positive or negative) shows moderate stability, higher than 40 mV (either positive or negative) resembles high stability^41^. In the supplemental material (S1-A) the peak at − 9.52 mV is due to the presence of epoxy, OH, and COOH functional groups, which show that the whole surface is negative and instable.

The surface charges/zeta potential of silica is − 9.9 mV and 26.4 mV due to the presence of silanol functional groups and the overall zeta potential is − 11.5 mV as shown in the supplemental material (S1-B) and Table 2. The zeta potential of the resultant composite is also negative over all charge surface area is − 11.5 mV. The appearance of a peak at − 30.9 peaks is due to the esterification reaction, which are related to Si–OH and COOH. The peak at 13.4 mV and 2.4 mV are related to hydroxyl and epoxy functional groups as shown in the supplemental material (S1-C). These results show the successful formation of silica monolith and graphene oxide composite. At adjustable pH the negative surface of silica and GO composite can be effectively utilized for better adsorption of pesticides and electrostatic assembly can be effectively realized through the interaction of heterogeneous charges. In addition to the hydroxyl group on the surface of silica the carboxyl, epoxy, and phenolic hydroxyl groups are abundant on the surface of graphene oxide interacting through van der Waals force and hydrogen bonding^42^.

**Table 2:** Zeta potential of graphene oxide, silica monolith, and silica anchored graphene oxide composite.

S. no	Samples	Zeta potential (mV)
1	Graphene oxide	− 9.52
2	Silica monolith	− 11.5
3	Silica anchored GO composite	− 9.18

### Fourier transform scanning electron microscopy

**Figure 5:**
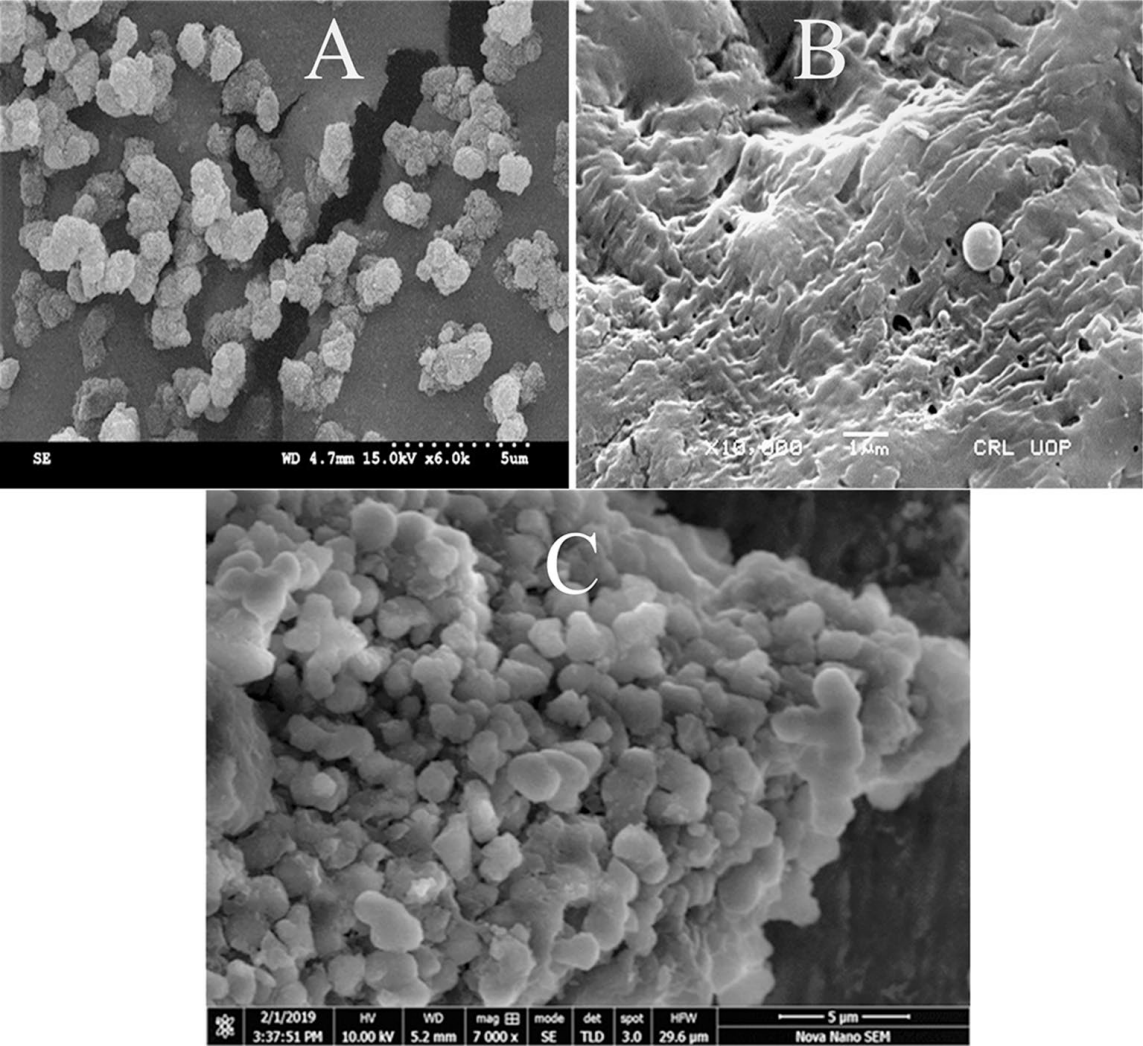
The FE-SEM images of Silica monolith (**A**), Graphene oxide (**B**), Silica anchored Graphene oxide composite (**C**).

The FE-SEM snap shots of Graphene oxide, silica monolith, and silica anchored graphene oxide composite were taken to check the surface morphology and architecture. The surface morphology and architectural plan of the bare graphene oxide and silica monolith and the associated changes after the incorporation of silica monolith into the graphene oxide suggests the synthesis of the composite. SEM image of the silica monolith given in Fig. 5A shows that the particle size of the silica monolith lies within 2 to 3 µm and the particles are rough with porous nature. Similarly, the SEM image of graphene oxide given in Fig. 5B depicts sheet like nature. The SEM image of the silica anchored graphene oxide is given in Fig. 5C where the growth of the silica particles on the surface of the graphene oxide can be clearly seen. Moreover, the surface porosity of the composite can be seen from the surface morphology of the SEM image of the composite.

### Batch adsorption

**Figure 6:**
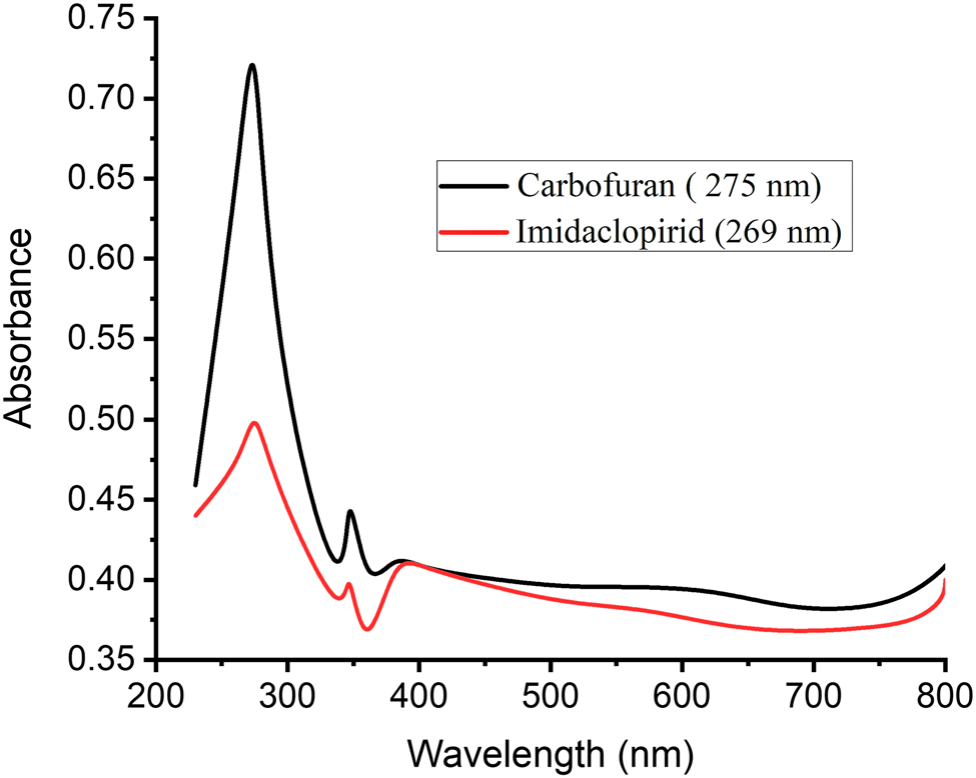
Wavelength of maximum absorbance for carbofuran and imidacloprid.

Adsorption of carbofuran and imidacloprid on the surface of Silica monolith anchored GO composite were studied spectrophotometrically. The wavelength of maximum adsorption for carbofuran ~ 275 nm and imidacloprid ~ 269 nm are shown in Fig. 6. Various parameters affecting the adsorptive removal of carbofuran and imidacloprid from wastewater were optimized.

### pH effect

**Figure 7:**
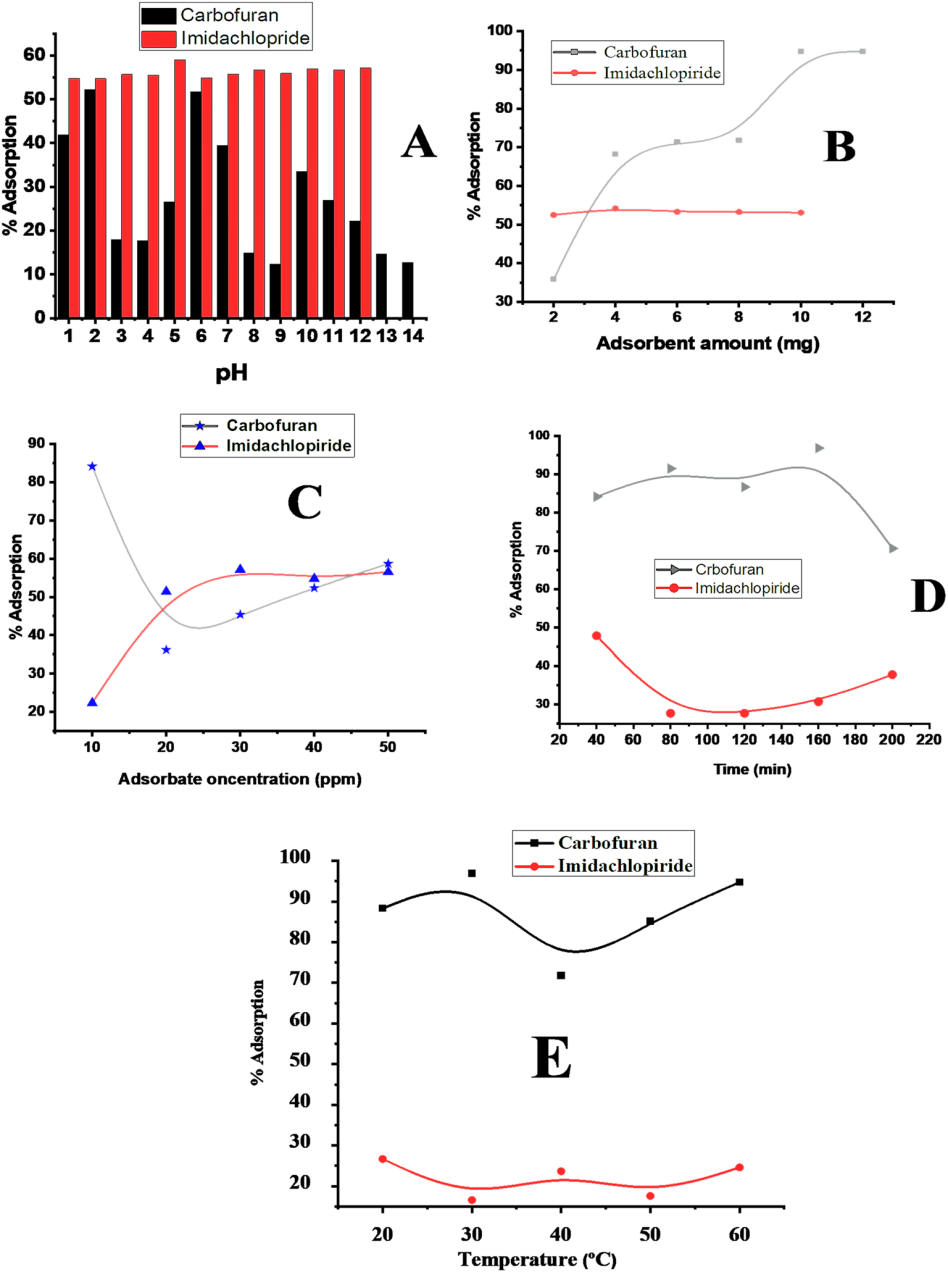
Effect on % adsorption of carbofuran and imidacloprid: pH (**A**); adsorbent dose (**B**); amount of adsorbate (**C**); shaking time (**D**); temperature effect (**E**) during the adsorption on composite surface.

Different pH solutions (1–14) were prepared keeping other conditions constant such as adsorbate concentration (20 ppm), adsorbent dose (15 mg), contact time (30 min) in 25 ml volume for carbofuran and imidacloprid. pH 5 was observed to be the optimum pH for imidacloprid as shown in Fig. 7A. According to the zeta potential of the composite (− 9.18 mV) the whole surface of the composite should be negative at pH below 9.18. At higher pH in acidic media, the concentration of hydrogen ion is limited and thus the positively charged (NH) of imidacloprid can easily undergo into the electrostatic interactions with the adsorbent. At pH lower than 5, the higher hydrogen ion concentration competes with the imidacloprid and thus the %adsorption of imidacloprid decreases at pH lower than five^43^. A slightly increase in the % adsorption was observed towards basic medium owing to the higher degree of dissociation of the silanol (Si–OH) groups into the Si–O^−^ which causes electrostatic interaction of NH functionalities of the imidacloprid with negatively charged composite^44^. For the adsorption of carbofuran maximum % adsorption was observed at pH 2 for a 10 ppm carbofuran solution by taking 6 mg of the adsorbent at room temperature having a contact time of 30 min in 25 ml solution as shown in Fig. 7A, at lower pH in acidic media, the surface site of the composite become positive due to protonation of the surface functionalities of the composite. The positively charged surface of the composite result in strong electrostatic interaction with the negatively charged functional groups of carbofuran. At elevated pH values, the protonated sites of the composite decreases which reduce the adsorption of carbofuran^45^.

### Effect of adsorbent dose

The adsorption capability of silica anchored graphene oxide composite towards imidacloprid and carbofuran was observed to be affected by changing the adsorbent dose as it alters the functional surface area of the adsorbent. A slight increase in the % adsorption of imidacloprid was observed by increasing the adsorbent dose from 0.2 mg L^−1^ to 0.8 mg L^−1^ with a maximum % adsorption of 53.304% at pH 5, contact time 40 min, adsorbate concentration 20 ppm, and solution volume of 10 ml at ambient temperature as shown in Fig. 7B. Similarly, the % adsorption of carbofuran was increased from 0.1 to 1 mg L^−1^ with a maximum % adsorption of 93.69% at pH 2, contact time 160 min, adsorbate concentration 20 ppm, and solution volume of 10 ml at ambient temperature as shown in Fig. 7B. The % adsorption of both imidacloprid and carbofuran were increased initially and then became constant at higher adsorbent dose. The occurrence of constant % adsorption beyond the optimized adsorbent amount indicates surface saturation of the adsorbent^46^.

### Effect of adsorbate

A series of carbofuran and imidacloprid solutions ranging from 10 to 50 ppm were prepared keeping other parameters constant. Maximum % adsorption of carbofuran (84.12%) was noticed at 10 ppm using adsorbent dose 0.8 mg L^−1^, pH 2, contact time 40 min in 10 ml of the solution at room temperature as shown in Fig. 7C. The % adsorption went on decreasing up to 20 ppm and then increased again. The % adsorption at lower adsorbate concentration (10 ppm) is maximum due to the availability of more active sites on the adsorbent surface. With the passage of time the active sites were occupied by the adsorbate molecules. Thus, the active sites per unit of the composite were diminished^47^. The increase in % adsorption at elevated concentration of carbofuran is due to film diffusion and particle diffusion. Film diffusion refers to the diffusion of adsorbate from external layer into the interior surface of the composite while the diffusion of adsorbate ions into the pores of the composite is the diffusion^48^. Maximum % adsorption of imidacloprid (57.169%) was observed at 30 ppm at the optimized conditions. Initially, the % adsorption of carbofuran was 23% at 10 ppm which increased linearly up to 57.169% at 30 ppm as shown in Fig. 7C. The increase in % adsorption by increasing adsorbate concentration is due to the availability of more adsorbate in bulk solution for adsorption. Higher adsorbate concentration helps to enhance the driving force to control all mass transfer resistance of adsorbate from bulk solution to the solid surface of the composite, which resulted in the increased the adsorbate-adsorbent interactions^49^.

### Shaking time

A series of five solutions of carbofuran and imidacloprid were prepared with 20 ppm concentration in 20 ml volumetric flask at the optimized constant conditions. All the solutions were shaken for different time interval (40–200 min) at the ambient temperature.

By shaking carbofuran the % adsorption increases linearly from 40 min up to 160 min and then decrease as shown in Fig. 7D. The increase in % adsorption by increasing the shaking time is since, the active sites of the composite participating in the interaction with carbofuran are crowded with passage of time and thus increasing % adsorption. After saturation of the active sites decrease in the % adsorption occurs as shown in Fig. 7D since some of the adsorbate molecules detach from the surface of the adsorbent with further increasing the shaking time^50^. Maximum % adsorption of imidacloprid (47.857) was found to be at 40 min as shown in the Fig. 7D. The observed maximum adsorption time is the time require for the achievement of adsorption equilibrium^51^.

### Effect of temperature

Temperature in varying degree can also affect the adsorption of carbofuran and imidacloprid on the surface of silica monolith anchored graphene oxide. A series of solutions of carbofuran and imidacloprid were prepared and the effect of temperature was observed by varying the temperature from 30 to 60 ℃ as shown in Fig. 7E. By increasing the temperature, the %adsorption of imidacloprid decreases which clearly depicts that the adsorption of imidacloprid on the composite is an exothermic process. Maximum %adsorption was found to be 26% at 20 ℃ for imidacloprid and 96.86% for carbofuran at 20 and 30 ℃ respectively as shown in Fig. 7E. The adsorption sensitivity of imidacloprid is greater at low temperature. The reason is that more adsorption site of lower energy participates in the adsorption of imidacloprid. The Freundlich parameter for the imidacloprid is the higher sensitivity to temperature^52^. Maximum %adsorption of carbofuran was found to be (96.86%) at 30 ℃. The increase in % adsorption was also observed above 40 ℃ due to the diffusion of adsorbate into the interior surface of the composite at high temperature. The decrease in % adsorption shows that the reaction is endothermic and by increasing temperature the activation energy of product decreases^53^^.^

### Isotherm study 

**Figure 8:**
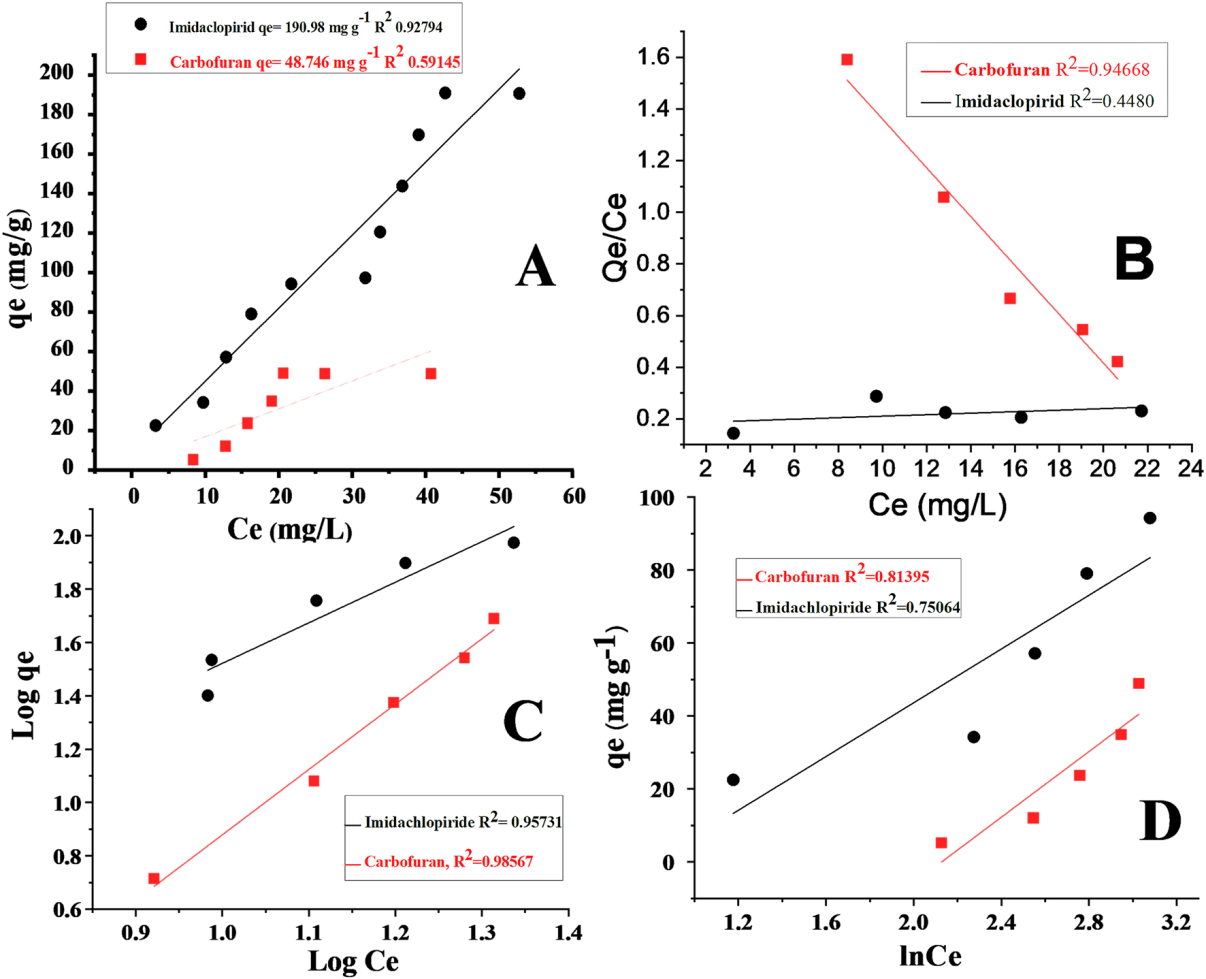
(**A**): General adsorption isotherm (**B**): Langmuir adsorption isotherm (**C**): Freundlich adsorption isotherm (**D**): Temkin adsorption isotherm for the adsorption of carbofuran and imidacloprid.

Different adsorption isotherms were applied to illustrate the equilibrium concentration of carbofuran and imidacloprid during their adsorption on the surface of silica anchored graphene oxide composite. The adsorption data at equilibrium was analyzed using the general adsorption isotherm (Fig. 8A) Langmuir (Fig. 8B), Freundlich (Fig. 8C), and Temkin models (Fig. 8D)^54,55^.

Langmuir adsorption isotherm depicts the monolayer adsorption on adsorbent surface having limited number of the active sites and uniform surface. The monolayer adsorption in the Langmuir equation is represented by qe which is 342.46 mg g^−1^ for imidacloprid and 37.27 mg g^−1^ for carbofuran. KL in L mg^−1^ is the constant of Langmuir adsorption related to the adsorption affinity of adsorbate to the binding sites. KL for the imidacloprid was 0.016 L mg^−1^ and 0.0409 L mg^−1^ for carbofuran^56^.

1 $$\frac{{ C_{e} }}{{Q_{e} }} = \frac{1}{{K_{L} Q_{m} }} + \frac{{C_{e} }}{{Q_{m} }}$$

2 $${\text{Log qe}} = \log {\text{KF}} + \frac{1}{{\text{n}}}\log {\text{Ce}}$$

3 $${\text{qe}} = {\text{BlnA}} + {\text{BlnCe}}$$

Freundlich adsorption isotherm is usually used to represent the adsorption of adsorbate on heterogeneous non-uniform surface of adsorbent^57^ as shown in Eq. 2. Freundlich adsorption constant KF (mg g^−1^) represent the adsorption capacity. The KF value was reported to be 1.019 mg g^−1^ for imidacloprid and 37.230 mg g^−1^ for carbofuran. Freundlich parameter “1/n” is heterogeneous and sorption factors. When 1/n < 1 (n > 1) shows the favorability of adsorption of pesticides on the surface of the composite. The values of 1/n for imidacloprid and carbofuran was calculated to be 0.657 and 0.4083 respectively^58^. The adsorption data of carbofuran and imidacloprid with regression values of 0.985 and 0.957 as shown in Fig. 8B and Fig. 8C respectively shows that they follow the Freundlich adsorption isotherm. The models illustrated in Fig. 8B and C show that the adsorbate-adsorbent interaction is due to the hydrogen bonding interaction between adsorbate and adsorbent. The hydrogen bond can favorably exist among hydrogen and the heteroatoms (oxygen and nitrogen) present in the adsorbate and adsorbent and thus the adsorption is physisorption. The lower regression values 0.448 ~ imidacloprid and 0.946 ~ carbofuran suggest that the Langmuir adsorption isotherm could not best explain the adsorbate-adsorbent interaction mechanism. Thus, the possibility of the electrostatic interaction among the negative functional groups of the composite and positive functional groups (NH^+^) of the pesticides seems very less.

Temkin adsorption isotherm was applied to the data. The linear form of Temkin is given in Table 3. along with constant parameters. Temkin constant (b) is related to the adsorption heat in J mol^−1^, R is gas constant (8.314 J mol^−1^ K^−1^), T is temperature (K) and A is the Temkin constant^59^. The values of constant A and b were noted from the intercept and slop of the plot of qe vs ln Ce as shown in Fig. 8D Table 3. The reasonable regression values 0.75 ~ imidacloprid and 0.812 ~ carbofuran because of the Temkin model application suggest that the diffusion was also involved during the adsorption process. The equations for Langmuir, Freundlich, and Temkin adsorption isotherms along with the calculated parameters and *R*^2^ values are given in Table 3.

**Table 3:** Adsorption isotherm models for the adsorption of carbofuran and imidacloprid on Silica monolith—GO composite.

Isotherm model	Langmuir model			Freundlich model				Temkin model		
Linear equation	Ce/qe = 1/KL Qm + Ce/Qm			Log qe = log KF + 1/n log Ce				Qe = B ln A + B ln Ce		
Parameters	KL (L g^−1^)	Qe (mg g^−1^)	*R*^2^	KF (mg g^−1^)	N	1/n	*R*^2^	A	B (J mol^−1^)	*R*^2^
Midacloprid	0.016	342.46	0.448	1.019	1.52	0.657	0.957	0.149	36.84	0.750
Carbofuran	0.040	10.582	0.946	37.23	2.45	0.408	0.985	0.007	44.98	0.812

### Kinetic adsorption studies

**Figure 9:**
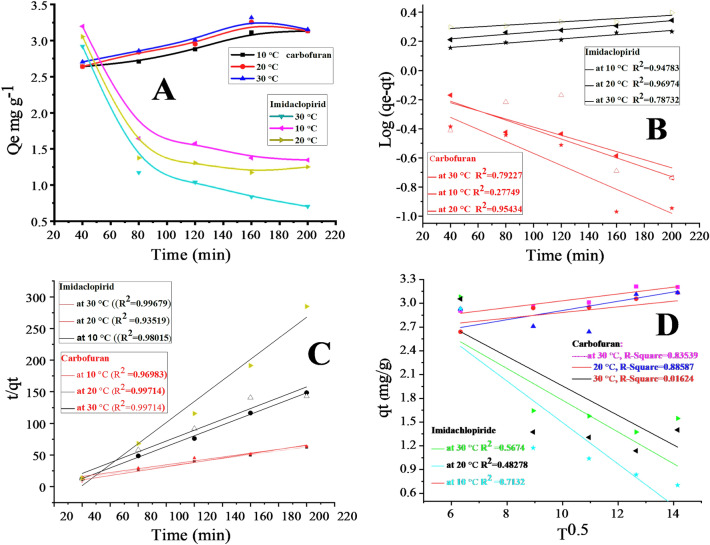
General adsorption kinetic model: (**A**), Carbofuran; at 10 ℃ (■), 20 ℃ (●), and 30 ℃ (▲); imidacloprid; 10 ℃ (◀), 20 ℃ (▶) and 30 ℃ (▼). Pseudo first order kinetic model: (**B**), imidacloprid; at 10 ℃ (☓), 20 ℃ (◀) and 30 ℃ (★), carbofuran; at 10 ℃ (∆), 20 ℃ (◀) and 30 ℃(★), Pseudo second order kinetic model: (**C**), imidacloprid; at 10 ℃ (△), 20 ℃ (▲) and 30 ℃ (●), carbofuran; at 10℃ (☓), 20 ℃ (▲) and 30 ℃(_×_) intra particle diffusion model: (D), carbofuran; at 10 ℃ (■), 20 ℃ (▲) and 30 ℃ (●) imidacloprid; at 10 ℃ (_×_), 20 ℃ (▲) and d 30 ℃ (▲) during the adsorption on the composite.

To study the adsorption mechanism and rate controlling step of imidacloprid and carbofuran on the surface of silica graphene oxide the kinetic study was demonstrated. Different temperature ranging from 10 to 30 ℃ and contact time from 40 to 200 min were used during the adsorption of carbofuran and imidacloprid on the surface of the composite. The amount adsorbed of carbofuran (Qe in mg g^−1^) initially increases with increasing time at different temperature (10 to 30 ℃) where the maximum value was obtained at 160 min and then became constant. Maximum Qe was found to be 3.317 mg g^−1^ at 30 ℃. A slight increase was observed during the adsorption of carbofuran by increasing temperature which showed that the adsorption is an endothermic process. The adsorbed amount (Qe in mg g^−1^) of imidacloprid was decreasing by increasing the contact time at 10–30 ℃. Maximum Qe (3.198 mg g^−1^) was found at the contact time of 40 min at 10 ℃. The results of kinetic study for carbofuran and imidacloprid are summarized in Table 4 and Fig. 9. Decreased in the Qe for imidacloprid at higher temperature indicates that the adsorption of imidacloprid is an exothermic process as shown in Fig. 9. The adsorption rate declined for carbofuran after 160 min and that of the imidacloprid after 90 min is mainly due to the decreasing of active sites of the composite for the adsorption and sluggish diffusion.

**Table 4:** Kinetic study for the adsorption of imidacloprid and carbofuran on silica anchored GO composite.

Pesticides	Temperature (℃)	Pseudo 1st order				Pseudo 2nd order				Intra particle diffusion		
		K1 (min^−1^)	Qe (mg g^−1^) (cal)	Qe (mg g^−1^) (exp)	*R*^2^	K2 (min^−1^)	Qe (mg g^−1^)(cal)	Qe (mg g^−1^) (exp)	*R*^2^	Kid (min^−1/2^)	C (mg g^−1^)	*R*^2^
Carbofuran	10	0.0064	1.2853	3.317	0.27	0.022	3.273	3.317	0.96	0.0356	2.5256	0.02
	20	0.0074	1.2052	3.317	0.95	1.926	3.330	3.317	0.99	0.0584	2.3248	0.88
	30	0.0094	1.435	3.317	0.79	1.926	3.331	3.317	0.99	0.0430	2.6008	0.83
Imidacloprid	10	0.0016	1.346	3.198	0.95	0.042	0.601	3.198	0.97	0.2619	4.113	0.71
	20	0.0017	1.5365	3.198	0.97	0.055	1.170	3.198	0.93	0.2007	3.7828	0.49
	30	0.0013	1.8451	3.198	0.79	0.339	1.180	3.198	0.99	0.1864	3.8209	0.57

The linear form of three kinetic models Pseudo first order (Eq. 4) pseudo second order (Eq. 5) and intra particle diffusion (Eq. 6) were applied to the adsorption data of imidacloprid and carbofuran.

4 $${\text{log }}\left( {{\text{qe }}{-}{\text{ qt}}} \right){ } = {\text{ log qe }}{-}{\text{ K}}1{ }\frac{{\text{t}}}{2.303}$$

5 $$\frac{{\text{t}}}{{\text{ qt}}} = \frac{1}{{{\text{k}}_{2} {\text{q}}_{{\text{e}}}^{{2{ }}} }} + \frac{{\text{t}}}{{{\text{q}}_{{\text{e}}} }}$$

6 $${\text{qt}} = {\text{k}}_{{{\text{id}}}} {\text{t}}^{{{1}/{2}}} + {\text{C}}$$

where K_1_ in min^−1^, K_2_ in g.mg^−1^.min^−1^, and Kp in mg^−1^.mn^−1^ are the rate constant for the pseudo first order, pseudo second order, and intra particle diffusion respectively. The qt and qe in mg.g^−1^ are the adsorbed amount of the pesticides at time (t) and equilibrium time respectively^60^. The linear form of all kinetic models is shown in Fig. 9 for both of the pesticides, while their related parameters have been given in Table 4.

In pseudo first order kinetic model the values of K1 and qe were determined from the slope and intercept of the plot [log (qe − qt) Vs time] at different temperature which are summarized in Table 4. In pseudo 2nd order the values of qe and K_2_ were determined from the slope and intercept of the plot (t/qt) against time at different temperatures as shown in Fig. 9C and Table 4.

In intra particle diffusion the plot of qt against T ^0.5^ shows the multi kinetic stages during adsorption process. The first stage is fast diffusion second is slow and the third is slowest step of diffusion into the pores of the composite^61^. The pseudo 2nd order for the adsorption data of carbofuran (*R*^2^ = 0.9970) is more applicable than that of the pseudo first order (*R*^2^ = 0.7922) and intra particle diffusion (*R*^2^ = 0.8353). The qe value calculated from the pseudo 2nd order model (3.331 mg g^−1^) and that calculated experimentally (3.317 mg g^−1^) at 20 ℃ are closely related to each other as shown in Table 4 and Fig. 9C. The higher *R*^2^ value and closeness of the qe values (calculated and experimental) suggests that the data of adsorption of carbofuran on the surface of composite follow pseudo second order kinetic model and the adsorption is physisorption^62^.

Similarly, for imidacloprid the higher *R*^2^ (0.9967) value favors the pseudo second order model in comparison to the pseudo first order model (*R*^2^ = 0.7873) and intra particle diffusion (*R*^2^ = 0.5674) at 30 ℃ given in Fig. 9D. The values of qe calculated from the equation and that determined experimentally are in agreement of the physisorption mode.

### Equilibrium and thermodynamic studies

**Figure 10:**
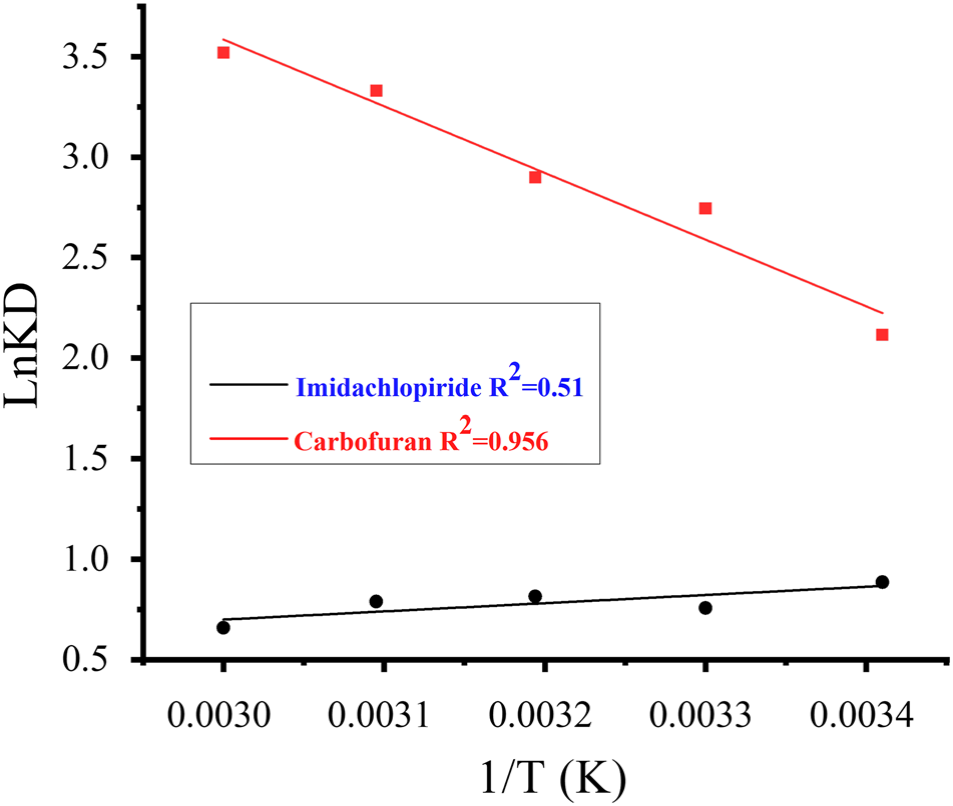
Van't Hoff equation for the adsorption of imidacloprid and carbofuran.

Thermodynamics and equilibrium parameters like change in entropy, enthalpy, and Gibbs free energy were calculated to determine the feasibility of adsorbate interaction with the composite. In conclusion the adsorption process is spontaneous in nature. Thermodynamic parameters such as ∆H, ∆S and ∆G were calculated using the following equation.

7 $$\Delta {\text{G}} = - {\text{RTlnKD}}$$

8 $$\begin{aligned} {\text{KD}} & = {\text{qe}}/{\text{Ce}} \\ \ln {\text{KD}} & = - \frac{{\Delta {\text{G}}}}{{{\text{RT}}}} = - \frac{{\Delta {\text{H}}}}{{{\text{RT}}}} + \frac{{\Delta {\text{S}}}}{{\text{R}}} \\ \end{aligned}$$

where qe is the adsorbed amount in mg.g^−1^, KD is distribution constant and Ce shows the equilibrium concentration in mg L^−1^. A plot of lnKD versus 1/T was constructed as shown in Fig. 10 for imidacloprid and carbofuran. The results of thermodynamic study (∆H, ∆S, and ∆G) are given in Table 5.

**Table 5:** Van't Hoff parameters for the adsorption of imidacloprid (■); and carbofuran (●); on Silica anchored GO composite.

T (k)	1/T(k)	Imidacloprid			Carbofuran		
		KD	lnKD	− ∆G KJ/mol	KD	lnKD	− ∆G KJ/mol
293	0.00341	2.42	0.685	1670.61	8.29	2.115	5154.03
303	0.00330	2.13	0.757	1906.99	15.54	2.743	6912.27
313	0.00319	2.32	0.815	2121.90	27.62	3.318	8635.93
323	0.00309	2.15	0.789	2119.28	7.060	1.954	5248.38
333	0.00300	2.36	0.858	2377.08	12.73	2.544	7043.22

The values of ∆H (kj mol^−1^) and ∆S (JK^−1^ mol^−1^) for imidacloprid are − 3400.42 and 4.323 and for carbofuran are 27,381.66 and 112.687 respectively.

The positive value of ∆H (27,381.66) for carbofuran shows that the reaction is endothermic. and the negative value of ∆H (− 3400.42) for imidacloprid shows that the reaction is exothermic. The positive value of ∆S for both carbofuran (112.687) and imidacloprid (4.323) indicate strong affinity of the pesticide’s adsorption^63^. The negative value of ∆G for both pesticides show spontaneous nature of adsorption and chemisorption as shown in Table 5.

### Application in real water sample

Application of the newly synthesized silica monolith anchored GO composite, for the removal of carbofuran and imidacloprid from real water sample is very important for the validation of the developed method. The removal of carbofuran and imidacloprid by silica anchored GO composite from three different samples was demonstrated. The samples were collected from distilled water obtained from the laboratory distillation plant, peach garden, and tomato garden from the local former field of Gulabad Distt., Dir KPK province Pakistan. The concentration of carbofuran and imidacloprid was measured in the samples prior to the pretreatment process resulting in the amount of the pesticides which are below the detection limit of the UV spectrophotometer. Therefore, all the samples were spiked by adding 100 ppm adsorbate concentration. The %adsorbed amount of both carbofuran and imidacloprid was calculated by comparing the pesticides concentration before and after adsorption. The percent adsorption of both pesticides from real water sample was calculated at the optimized conditions. The amount of the carbofuran was 99.77% in distilled water, 86.538% in peach garden sample, and 83.81% in tomato garden sample for carbofuran. The percent adsorption of imidacloprid was found to be 99.18% in distilled water, 91.579% in peach garden, and 90.066% in tomato garden real water sample as given in Table 6. The observed difference in percent adsorption in different water sample may be due to the difference of pH and the presence of other contamination which suppress the adsorption of carbofuran and imidacloprid in peach and tomato garden samples^64^.

**Table 6:** Removal of the pesticides from real water samples.

Samples	Carbofuran		Imidacloprid	
	Added amount (ppm)	% Adsorption	Added amount (ppm)	% Adsorption
Distilled water	100	99.77	100	99.18
Peach garden	100	86.538	100	91.579
Tomato garden	100	83.81	100	90.066

The comparison of the adsorption capacity of the composite (current study) with those of the adsorbents reported in literature has been summarized in Table 7. The Table shows that the absorption capacity of the adsorbent of current study (for carbofuran and imidacloprid) is much higher those of the adsorbents reported in literature.

**Table 7:** Comparison of adsorption capacity of the composite of current study with those of the adsorbents reported in literature.

Adsorbent	Adsorbate	Adsorbate (ppm)	Adsorbent (mg)	Adsorption capacity (mg-g^−1^)	References
Chitosan-based	Basic blue 3 dye	20	40	166.5	^65^
Durian husk	Basic blue 3	50	100	49.5	^66^
Coconut charcoal	Monocrotophos (pesticide)	10	200	103.9	^67^
Ac. carbon of hyacinth	Glyphosate (pesticide)	100	3	240.8	^68^
Chitin	Atrazine	20	20	113.23	^69^
Acrylic resin	Basic blue 3	100	50	46.95	^70^
Peat	Basic dyes	200	4	41.00	^71^
Silica anchored GO	Imidacloprid	30	4	342.46	Current study
Silica anchored GO	Carbofuran	10	10	37.23	Current study

## Conclusions

Silica anchored graphene oxide composite was synthesized by the modified Fischer esterification protocol. The composite was used as an efficient adsorbent for the removal of carbofuran and imidacloprid from wastewater and real water sample. The characterizations such as XRD, FTIR, BET/BJH analysis, and Zeta potential were carried out for the newly synthesized composite which confirmed the synthesis of silica anchored graphene oxide composite. The functional groups such as C–O–C, COOH, OH were confirmed by FTIR. While the surface charge was determined using the zeta potential. 97% adsorption was achieved for carbofuran at pH 2, contact time = 1 h, adsorbate concentration = 10 ppm, adsorbent amount = 6 mg while 99% adsorption was observed for imidacloprid at pH 5, contact time = 1 h, adsorbent dose = 3 mg, and adsorbate amount = 20 ppm. The demonstrated Kinetic studies went in favor of the pseudo 2nd order kinetic model for carbofuran (*R*^2^~0.9971) and imidacloprid (*R*^2^~0.9967). Similarly, carbofuran and imidacloprid follows Freundlich adsorption isotherm with *R*^2^ = 0.995 for carbofuran and *R*^2^ = 0.950 for imidacloprid. The thermodynamic parameter such as change in enthalpy (∆H) for carbofuran is positive, which shows the endothermic and non-spontaneous nature of the adsorption process, while that of imidacloprid (∆H) is negative which shows the exothermic and spontaneous nature of adsorption. The positive change in entropy (∆S) represent the adsorption affinity of imidacloprid and carbofuran towards the silica anchored graphene oxide composite.

## Supplementary Information

Supplementary Information.

## Data Availability

The datasets generated and/or analyzed during the current study are not publicly available because we will use the same datasets in combination with other datasets for our future research but are available from the corresponding author on reasonable request.

## Abbreviations

AChE: Acetylcholinesterase
PEG: Polyethylene glycol
GO: Graphene oxide
AOP: Advance oxidation process
BET: Brunauer, Emmett and Teller
TMOS: Trimethoxy silane
ASTM: American society for testing and materials
