# Supplementary material for: Synthesis of monolith silica anchored graphene oxide composite with enhanced adsorption capacities for carbofuran and imidacloprid
Source: Sci Rep. 2022 Dec 5;12:21027. doi: 10.1038/s41598-022-25528-0 (PMC9722712; doi:10.1038/s41598-022-25528-0)
Supplement: Supplementary file 1 — Supplementary Information. [file 41598_2022_25528_MOESM1_ESM.docx]

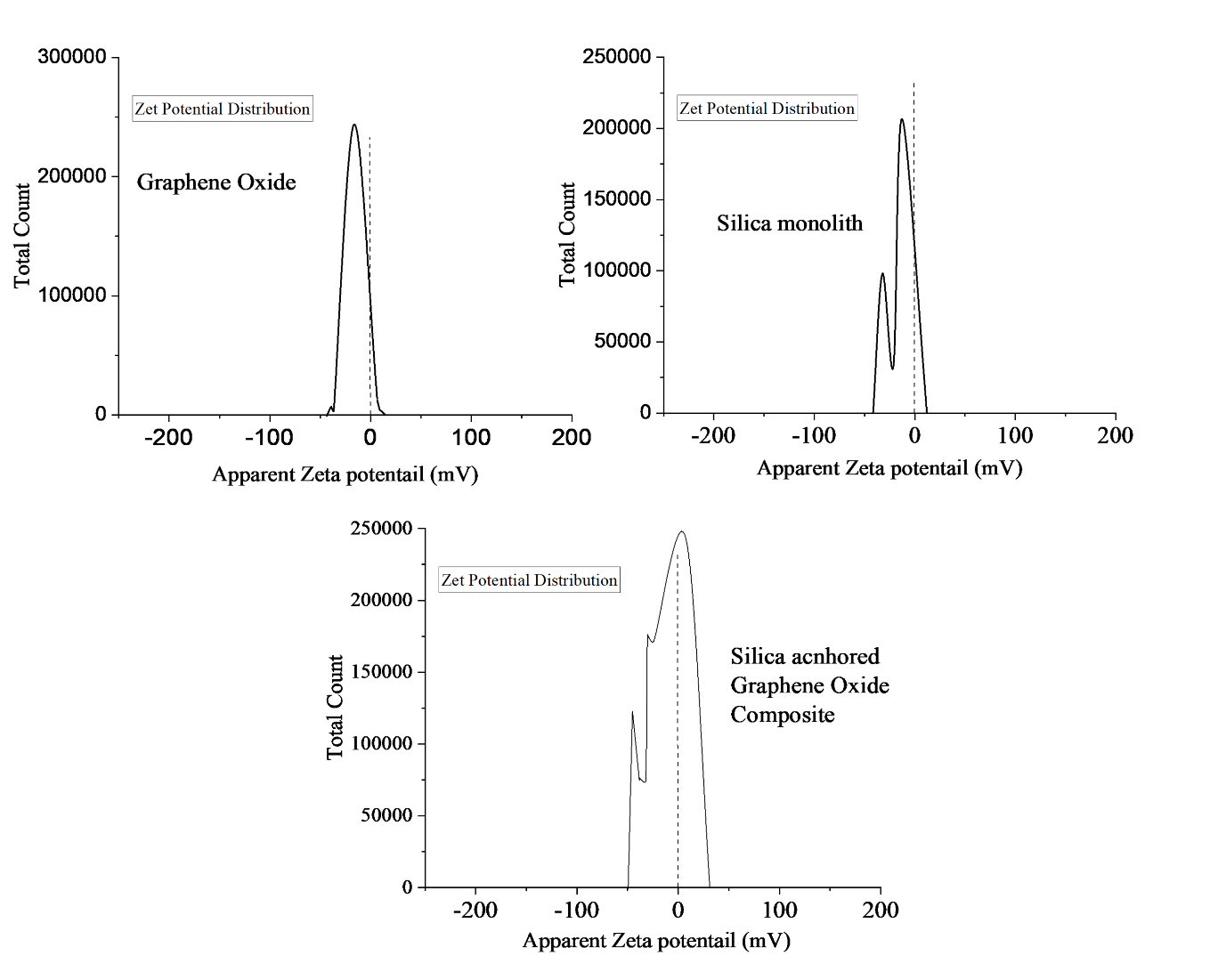


Supplemental materials (S_1_). zeta potential of graphene oxide (A) silica monolith (B), and silica anchored graphene oxide.
